# Migration Patterns and Potential Risk Assessment of Trace Elements in the Soil–Plant System in the Production Area of the Chinese Medicinal Herb *Scrophularia ningpoensis* Hemsl.

**DOI:** 10.3390/toxics12050355

**Published:** 2024-05-11

**Authors:** Yufeng Gong, Wei Ren, Zhenming Zhang

**Affiliations:** 1 School of Pharmacy, Guizhou University of Traditional Chinese Medicine, Guiyang 550025, China; gs.ycjiang22@gzu.edu.cn (Y.G.); gs.yangxy21@gzu.edu.cn (W.R.); 2 College of Resources and Environmental Engineering, Guizhou University, Guiyang 550025, China; 3 School of Resource and Environmental Engineering/Key Laboratory of Karst Geological Resources and Environment, Ministry of Education, Guizhou University, Guiyang 550025, China; 4 Field Scientific Observatory of Karst Environment and Ecosystem, Ministry of Education, Guiyang 550025, China

**Keywords:** trace element, risk assessment, *Scrophularia ningpoensis* Hemsl., Chinese herbal medicines

## Abstract

*Scrophularia ningpoensis* Hemsl. holds a prominent place among Chinese medicinal herbs. Assessing the soil–plant system of its origin is crucial for ensuring medication safety. Although some trace elements are essential for the normal functioning of living organisms, exposure to higher concentrations is harmful to humans, so in order to assess the possible health risk of trace elements in the soil–plant system of *Scrophularia ningpoensis* Hemsl. origin for human assessment, we used non-carcinogenic risk (HI) and carcinogenic risk (CR) for their evaluation. In this paper, the following trace elements were studied in the soil–*Scrophularia ningpoensis* Hemsl. system: manganese (Mn), iron (Fe), cobalt (Co), zinc (Zn), selenium (Se), molybdenum (Mo), arsenic (As) and lead (Pb). Correlation and structural equation analyses showed that the effect of soil in the root zone on the plant was much greater than the effect of soil in the non-root zone on the plant. The single-factor pollution index (Pi) showed that the soil in the production area of *Scrophularia ningpoensis* Hemsl. was polluted to a certain extent, notably with Pb showing the highest average Pi values of 0.94 and 0.89 in the non-root and root zones, respectively. Additionally, the Nemerow composite pollution indices (PN) for both zones indicated an alert range. Regarding health risks, exposure to soil in the non-root zone posed higher non-carcinogenic risk (HI) and carcinogenic risk (CR) compared to the root zone, although neither zone presented a significant carcinogenic risk. The potential non-carcinogenic risk (HI) and carcinogenic risk (CR) from consuming *Scrophularia ningpoensis* Hemsl. leaves and stems were more than ten times higher than that of roots. However, the carcinogenic risk (CR) values for both the soil and plant of interest in the soil– *Scrophularia ningpoensis* Hemsl. system did not exceed 10^−4^, and therefore no significant carcinogenic risk existed.

## 1. Introduction

Trace elements serve as the essential building blocks necessary for the normal growth and development of both plants and animals. They actively partake in critical material metabolic processes within organisms, contributing significantly to vital physiological and biochemical functions [1,2]. However, some of the trace elements are highly toxic to humans, such as arsenic and lead. Heavy exposure to these toxic elements can have negative effects on human health, such as cancer and neurological damage [3]. Lead can accumulate in the gray matter of the brain, damaging not only neurons and their dendrites and synapses, but also red blood cells [4]. In addition, arsenic and lead may also affect DNA and its regulatory mechanisms (IARC) and are therefore classified as known or probable human carcinogens [5]. It has been shown that moderate concentrations of zinc can improve seed germination, but high concentrations of zinc can inhibit plant growth [6]. Moderate amounts of iron can prevent or hinder protein pathology [7]. When manganese is deficient in animals, it interferes with normal growth rates [8]. Molybdenum may interfere with the balance of calcium and phosphorus in the animal’s body, leading to bone damage [9]. Overall, some of the micronutrients act as essential nutrients for plants and animals and they are important for their physiological and biological functions; however, their increased intake above certain permissible limits may be toxic [10].

The production of Chinese herbal medicines significantly contributes to the global agricultural economy, witnessing a consistent increase in market demand each year. These herbs possess the ability to absorb pollutants, either from the soil through their roots or from the air by accumulating pollutants on their leaves [11,12]. Human consumption of these contaminated Chinese herbs elevates the risk of diseases. *Scrophularia ningpoensis* Hemsl. stands as a widely used Chinese herbal medicine, with people utilizing its dried root as an anti-inflammatory agent to address fever, swelling, sore throat, and constipation [13,14,15]. Due to its high medicinal value, the market demand for *Scrophularia ningpoensis* Hemsl. has been consistently rising. However, the relentless pursuit of high yields and efficiency in agricultural production, alongside environmental factors, renders crops vulnerable to contamination by trace elements, aflatoxins, and pesticides [16]. Specifically, trace element pollution leads to a decline in crop quality, posing serious threats to both human lives and health, as well as the ecological environment [17]. Furthermore, it adversely impacts the wholesome development of the Chinese herbal medicine industry. Consequently, evaluating the health risks associated with trace elements present in the soil and plants originating from *Scrophularia ningpoensis* Hemsl. becomes exceptionally crucial.

Numerous studies have evaluated the risks associated with human exposure to trace elements in soil and crops across various countries [18,19,20]. However, there is a notable absence of studies addressing the concurrent risk of human exposure to trace elements in soil and Chinese herbal medicines. In papers on risk evaluation, there are fewer studies that talk about the root zone and non-root zone, so we measured soil samples from the non-root zone and the root zone to discuss the differences between the two. At the same time, we used structural equation modeling to explore the relationship between trace elements in different tissue sites in the soil–*Scrophularia ningpoensis* Hemsl. system. Building upon this gap, the current study aimed to investigate trace elements present in soils and *Scrophularia ningpoensis* Hemsl. plants sourced from production areas, with specific objectives: (1) to assess the contamination status of trace elements (Mn, Fe, Co, Zn, Se, Mo, As, and Pb) in *Scrophularia ningpoensis* Hemsl. production areas; (2) to evaluate the accumulation of trace elements in *Scrophularia ningpoensis* Hemsl. tissues (roots, stems, and leaves), alongside an analysis of potential health risks posed by these trace elements to consumers; and (3) to explore the transport and transformation characteristics of trace elements within the soil–*Scrophularia ningpoensis* Hemsl. system. The outcomes of this study are expected to provide valuable insights for optimizing planting structures within Chinese herbal medicine production areas.

## 2. Materials and Methods

### 2.1. Overview of the Study Area

The study area encompasses Daozhen County in Southwest China, positioned in the northern region of Guizhou Province. It resides within a slope area that marks the transition from the Yunnan–Guizhou Plateau to the Sichuan Basin, situated between 107°21′–107°51′ E longitude and 28°36′–29°13′ N latitude. Daozhen County spans a jurisdictional area of 2157 square kilometers and features a landform characterized by solifluction, erosion, low-mountain peaks, and troughs. The region predominantly exhibits carbonate rocks, representing a typical example of karst geomorphology.

The climate in this area falls under the subtropical humid monsoon category, offering mild winters without severe cold and comfortable summers without scorching heat. The average annual temperature stands at 16 °C, accompanied by an annual rainfall ranging between 800–1400 mm [21].

### 2.2. Sample Collection and Pretreatment

**Figure 1:**
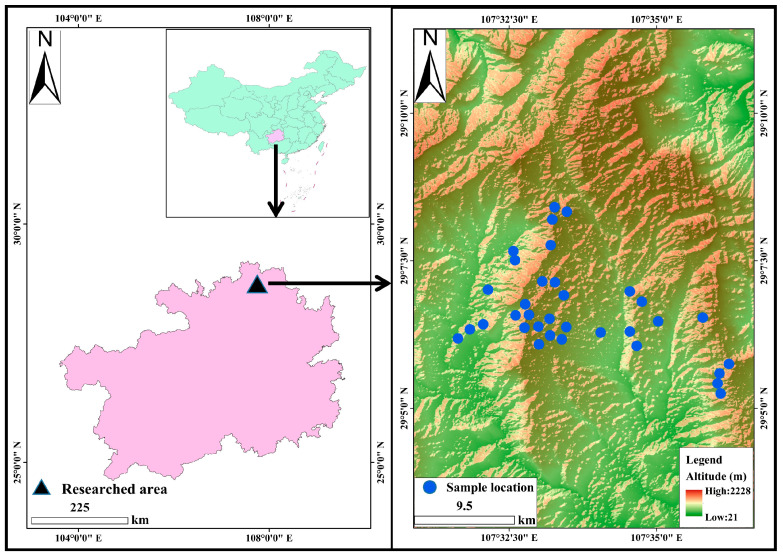
Map of the study area.

In Daozhen County, Southwest China, a representative and typical production area of *Scrophularia ningpoensis* Hemsl. was chosen for sampling. Soil samples were gathered from both the root zone and non-root zone, with an overview of the sampling site depicted in Figure 1. GPS was used to record the location of sampling points. A total of 68 soil surface samples (0–20 cm) were collected using the quadrat method, of which 34 were from the non-root zone and 34 from the root zone. Root zone samples were collected from within a 0–20 cm radius of the stem, while non-root zone samples were collected from within a 20–40 cm radius around the stem of *Scrophularia ningpoensis* Hemsl. Meanwhile, 102 samples of *Scrophularia ningpoensis* Hemsl. were collected correspondingly, including 34 roots, 34 stems and 34 leaves. The collected soil samples and *Scrophularia ningpoensis* Hemsl. plant samples were pre-treated and then analyzed for testing. Randomness was noted throughout the collection process. Soil trace elements (Mn, Fe, Co, Zn, Se, Mo, As, and Pb) were dissolved using a mixture of 5 mL HF, 8 mL HNO_3_, and 2 mL HCIO_4_. Trace elements within *Scrophularia ningpoensis* Hemsl. plants were digested using 8 mL HNO_3_ and 2 mL HCIO_4_. Soil and plant samples were digested as follows: 80 °C for 10 min—100 °C for 20 min—120 °C for 30 min—150 °C for 60 min—180 °C for 90 min—180 °C for 90 min—continuously 180 °C to a transparent color (1~2 mL left over from the digestion), cooled down, removed and poured into a glass digestion tube with deionized water and fixed to a scale of 50 mL to be measured. Each experiment was conducted in duplicate (standard deviation < 5%), and three reagent blanks were included. Three standard substances were used for both plants and soil, with soil standard reference material being GBW07405a (GSS-5a) (https://www.ncrm.org.cn/English/CRM/pdf/GBW07405_20160301_135939974_1704880.pdf, accessed on 25 April 2024) and plant standard reference material being GBW10015 (GSB-6) (https://www.ncrm.org.cn/English/CRM/pdf/GBW10015_20160301_143135112_1756484.pdf, accessed on 25 April 2024). The recovery rate of standard substances ranged from 90.3% to 105.6%. The trace elements in soil and plants were digested in a graphite digestion furnace [Haineng Future Technology Group Co. (SH230N)] and the trace element content in soil and plants was determined using inductively coupled plasma mass spectrometry ICP-MS [Perkin Elmer (NexION 2000)]. Samples for determination of ICP-MS are analyzed at room temperature with less than 5% acidity, Gas purity: helium (purity ≥ 99.999%) and argon (purity > 99.996%), Analysis speed: Analysis speed: >20 samples/hour. The carrier gas flow rate was 1.17 L/min, the gas mixture flow rate was 5.0 mL/min, the plasma RF power was 1300 W, the plasma flow rate was 15.0 L/min, the temperature of the atomization chamber was 2 °C, the integration time was 0.1 s, and the peristaltic pump speed was 30 r/min.

### 2.3. Evaluation Methodology

#### 2.3.1. Bioconcentration Factor (BF)

In this study, the bioconcentration factor (BF) was used to characterize the enrichment ability of *Scrophularia ningpoensis* Hemsl. plants for soil trace elements, which was calculated as [22]

(1) $$BF=C_{plant}/C_{soil}$$

where C_plant_ represents the heavy metal content in plant (mg.kg^−1^, dry weight) and C_soil_ represents the total heavy metal content in the corresponding soil sample (mg.kg^−1^). In this study, because two types of soils, root zone and non-root zone, were involved, we used root zone soils, which are in direct contact with plants, to calculate the bioconcentration factor (BF).

#### 2.3.2. The Single Factor Pollution Index (P_i_) and Nemerow Integrated Pollution Index (PN)

The single factor pollution index (P_i_) was used to assess the heavy metal contamination status of these *Scrophularia ningpoensis* Hemsl. soils [23]. The equation is as follows:

(2) $$P_{i}=C_{i}/S_{i}$$

where C_i_ is the individual heavy metal concentration in soil (mg kg^−1^) and S_i_ is the reference concentration of each metal in soil (risk screening values for heavy metal contamination of agricultural land in China, GB 15618-2018 https://www.mee.gov.cn/ywgz/fgbz/bz/bzwb/trhj/201807/t20180703_446029.shtml, accessed on 25 April 2024, pH > 5.5). To evaluate the overall contamination status of the *Scrophularia ningpoensis* Hemsl. soil samples, the Nemerow integrated pollution index (PN) was used in the present research. The PN can be calculated as follows:

(3) $$PN={\left[(P_{imax}^{2}+P_{iave}^{2})/2\right]}^{1/2}$$

where P_imax_ is the maximum value of P_i_ of all heavy metals considered in the present research and P_iave_ is the average value. The pollution level was categorized into clean (P_i_ ≤ 1.00), mildly polluted (1.00 < P_i_ ≤ 2.00), moderately polluted (2.00 < P_i_ ≤ 3.00), and heavily polluted (P_i_ > 3.00) based on the size of P_i_. The pollution level was categorized into safe (PN ≤ 0.70), alert limit (0.70 < PN ≤ 1.00), light pollution (1.00 < PN ≤ 2.00), medium pollution (2.00 < PN ≤ 3.00) and heavy pollution (PN > 3.00) based on the size of PN [24].

#### 2.3.3. Health Risk Assessment Indices

As for health risk assessment along the pathway of “soil-to-human”, we calculated the direct intake of soil via ingestion, D_S_ (mg·TE·kg^−1^·BW·d^−1^):

(4) $$D_{S}=C_{S}\cdot \frac{IngR\cdot EF\cdot ED}{BW\cdot AT}\cdot 10^{-6}$$

where IngR = Ingestion rate (100 mg·d^−1^); EF = Exposure frequency (350 d·yr^−1^); ED = Exposure duration (30 yr); BW = Body weight (70 kg); AT = Averaging time (ED × 365 d = 10,950 d); 10^−6^ is for unit conversion.

Subsequently, the non-carcinogenic risk was quantified with the use of the Hazard Quotient (HQ):

(5) $${HQ}_{S}=D_{S}/{RfD}_{0}$$

where RfD_0_ (mg·TE·kg^−1^·BW·d^−1^) is the reference dose of oral human intake. The values of RfD_0_ values used in this work are shown for each studied TE in Appendix A.

Also, HI, accounting for the corporate risk of all TEs combined:

(6) $${HI}_{S}=\sum \left({HQ}_{S}\right)$$

where HQ_S_ is the risk of all studied soil TEs. It must be noted that HQ > 1 indicates significant risk to human health, while HQ < 1 indicates that TE intake is not harmful. Similar is the case for HI. Human intake related to food consumption, D_F_ (units, mg·TE·kg^−1^·BW·d^−1^), was calculated as follows:

(7) $$D_{F}=C_{F}\cdot \frac{MIDVC\cdot EF\cdot ED}{BW\cdot AT}\cdot 10^{-6}$$

where C_F_ is TE concentrations in food (units mg kg^−1^ fresh food), and MIDVC is the mean individual daily food consumption, taken equal to 600 (mg·d^−1^) [25]. 

Further, we calculated food-induced Hazard Quotient (HQ_F_):

(8) $${HQ}_{F}=D_{F}/TDI$$

where TDI is the element maximum tolerable daily intake (units, mg·TE·kg^−1^·BW·d^−1^). The values for TDI used in this work are reported in Appendix A [26].

Also food-related Hazard Index (HI_F_) was calculated in order to account for the total risk of all individual TEs combined, as follows:

(9) $${HI}_{F}=\sum \left({HQ}_{F}\right)$$

#### 2.3.4. Indices Related to Carcinogenic Risks 

Cancer risk (CR, unitless) is based on the human intake values. For soil-related CR,

(10) $${CR}_{S}=C_{S}\cdot \frac{IngR\cdot EF\cdot LT}{BW\cdot AT}\cdot 10^{-6}\cdot OSF$$

where OSF is the oral (e.g., ingestion) slope factor. The OSF for As and Pb used in the our study were 1.5 and 0.0085 (mg·kg^−1^·day^−1^)^−1^. LT = Lifetime (70 yr) [26].

For food-related CR,

(11) $${CR}_{F}=C_{F}\cdot \frac{MIDVC\cdot EF\cdot LT}{BW\cdot AT}\cdot 10^{-6}\cdot OSF$$

A CR is considered acceptable if value is lower than 10^−6^, while the risk is significant when CR is higher than 10^−4^.

### 2.4. Data Analysis

The experimental data underwent pre-processing using Excel 2016 and subsequent analysis using SPSS 19.0. Analysis of variance was conducted, and multiple comparisons were carried out using the LSD method at a significance level of *p* < 0.05. The data were visualized using Origin 2022, RStudio 2022, and ArcGIS 10.8.

## 3. Results

### 3.1. Characteristics of Soil Trace Element Content in Root and Non-Root Areas of Scrophularia ningpoensis Hemsl. Origin

**Figure 2:**
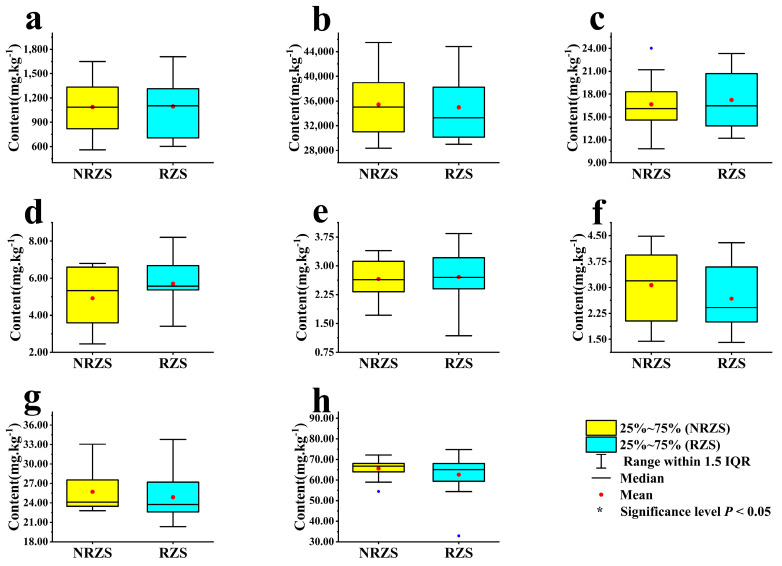
Characteristics of soil trace element contents in non-root and root zones. Note: Figures (**a**) (Mn), (**b**) (Fe), (**c**) (Co), (**d**) (Zn), (**e**) (Se), (**f**) (Mo), (**g**) (As), and (**h**) (Pb) represent examined the trace elements. NRZS denotes non-root zone soil, and RZS denotes root zone soil.

Distinct differences were observed in the characteristics of soil trace element content within *Scrophularia ningpoensis* Hemsl. production areas (refer to Figure 2). The quantities of Fe, Mo, As, and Pb elements exhibited higher levels in the root zone compared to the non-root zone. Conversely, Mn, Co, Zn, and Se displayed higher concentrations in the non-root zone compared to the root zone. However, these variations did not achieve statistical significance at the *p* < 0.05 level for all elements.

### 3.2. Characteristics of Trace Element Contents in Roots, Stems, and Leaves of Scrophularia ningpoensis Hemsl. Plants

**Figure 3:**
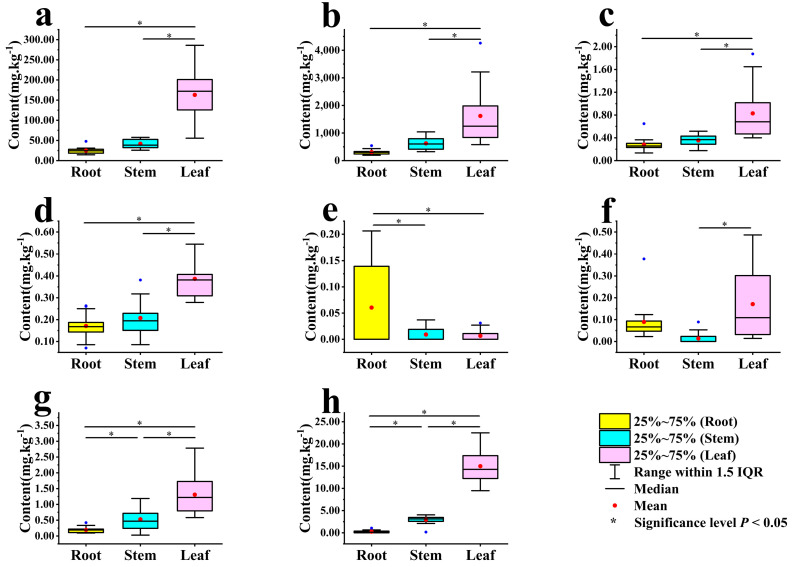
Characteristics of trace elements in roots, stems, and leaves of *Scrophularia ningpoensis* Hemsl. Plants. Note: Figures (**a**) (Mn), (**b**) (Fe), (**c**) (Co), (**d**) (Zn), (**e**) (Se), (**f**) (Mo), (**g**) (As), and (**h**) (Pb) represent examined the trace elements.

Significant variations were observed in the trace element contents across different parts of *Scrophularia ningpoensis* Hemsl. plants (refer to Figure 3). The content levels of Mn, Fe, Co, Zn, As, and Pb exhibited a pattern of leaf > stem > root. Specifically, the leaf content was markedly higher than both stem and root levels (*p* < 0.05). Notably, the root and stem contents displayed significant differences only in As and Pb levels (*p* < 0.05), Se was significantly higher in roots than in stems and leaves (*p* < 0.05).

### 3.3. Enrichment Factor (BF) of Roots, Stems, and Leaves of Scrophularia ningpoensis Hemsl.

Table 1 displays the bioconcentration factor (BF) of roots, stems, and leaves of *Scrophularia ningpoensis* Hemsl. Notably, Mn, Zn, and Mo exhibited the strongest enrichment effect in the roots, with an average BF of 0.03. Conversely, Pb demonstrated a weaker effect, nearly averaging a BF of 0. The highest BF for Pb in the stems and leaves of *Scrophularia ningpoensis* Hemsl. were 0.05 and 0.25, respectively. Furthermore, the enrichment coefficients of elements in leaves were generally higher compared to those in roots and stems, except for Mo. This indicates that, excluding Mo, the enrichment coefficients of all elements in leaves surpassed those in roots and stems, signifying the strongest enrichment effect in leaves.

### 3.4. Analysis of Single-Factor and Combined Pollution in Scrophularia ningpoensis Hemsl. Soils

**Figure 4:**
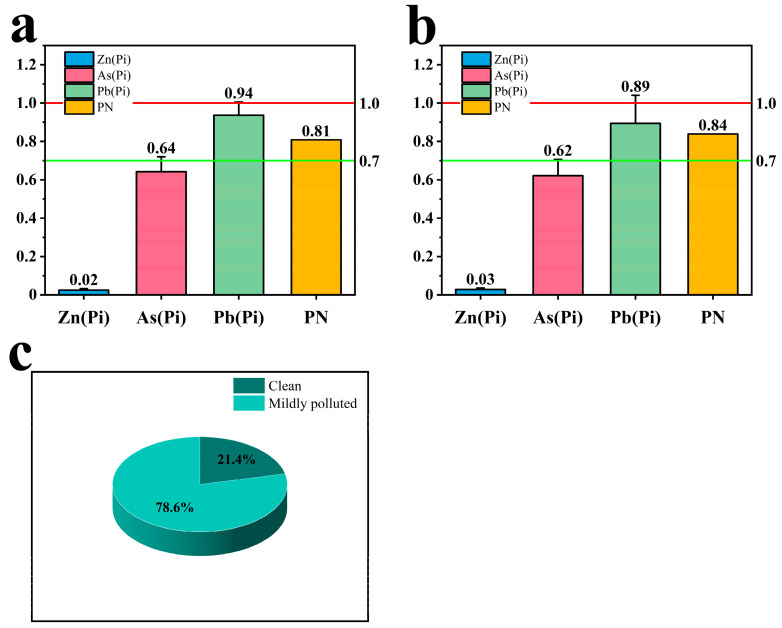
Single-factor pollution index (Pi) and combined pollution index (PN) of soil in non-root and root zones. Note: Figures (**a**,**b**) depict non-root zone and root zone soils, respectively. Figure (**c**) illustrates the percentage of contaminated soil.

This study calculated the single-factor pollution index (Pi) for three trace elements—Zn, As, and Pb—in the soil. The findings revealed that the average Pi values for these three trace elements were below 1.0. Notably, among them, Pb exhibited the highest average Pi values in both non-root (0.94) (Figure 4a) and root zones (0.89) (Figure 4b). For each of the three soil samples in the non-root and root zones, Pi values for Pb exceeded 1.0, indicating contamination in these specific samples. The contaminated sample percentage reached 21.4% (Figure 4c), suggesting a certain degree of soil contamination in the *Scrophularia ningpoensis* Hemsl. origin.

The combined pollution index (PN) for the non-root and root zone soils were 0.81 and 0.84, respectively. These values surpassed 0.7 but remained under 1.0, falling within the warning range. While not indicating severe trace element pollution, the values warrant attention, signaling a situation worthy of caution.

### 3.5. Hazard Coefficients (HQ) for Trace Elements in the Soil–Scrophularia ningpoensis Hemsl. System

The HQ_S_ and HQ_F_ values of trace elements are shown in Table 2. In our study, among the individual trace elements contributing to soil-related risks, it was observed that HQ_S_ for As ranked the highest in both the non-root and root zones (1.17 × 10^−4^ and 1.14 × 10^−4^, respectively). Regarding food-related risks, HQ_F_ for Fe dominated in roots and stems (3.63 × 10^−6^ and 7.33 × 10^−6^, respectively), while HQ_F_ for Pb was notably higher in leaves (3.52 × 10^−5^).

### 3.6. Evaluation of Non-Carcinogenic Risk in Scrophularia ningpoensis Hemsl. Soil and Plant

**Figure 5:**
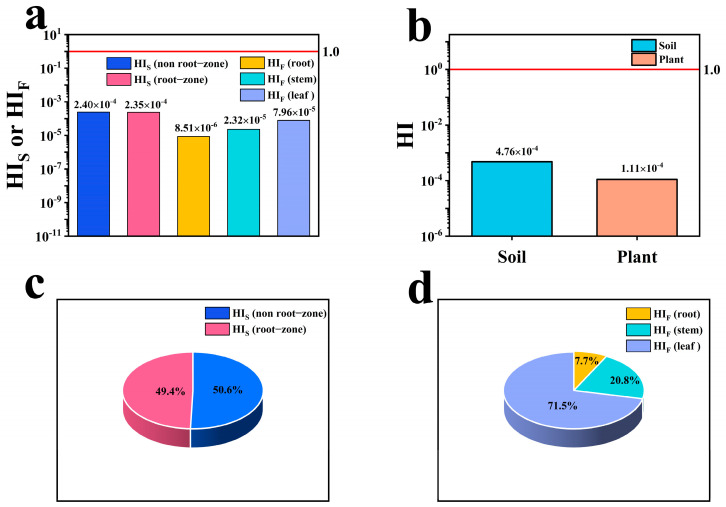
Hazard indices for direct soil exposure and food uptake associated with non−carcinogenic human health risks of trace elements in *Scrophularia ningpoensis* Hemsl. Production Areas. Note: Figure (**a**) represents the health risk (HI) values for soil and *Scrophularia ningpoensis* Hemsl. plants (roots, stems, and leaves) in the non−root zone and root zone, Figure (**b**) illustrates the total health risk (HI) values for soil and *Scrophularia ningpoensis* Hemsl. plants, while Figures (**c**,**d**) indicate the percentage of health risk (HI) in soil and plants, respectively.

The health risk (HI) values for soil in both the non-root and root zones, and across various parts (roots, stems, and leaves) of the *Scrophularia ningpoensis* Hemsl. plant from its origin, displayed variations (Figure 5a). In the soil, the HI value was higher in the non-root zone (2.40 × 10^−4^) than the root zone (2.35 × 10^−4^), with the non-root zone accounting for 50.6% of the total HI value of the soil (Figure 5c). For the plants, leaves (7.96 × 10^−5^) exhibited the highest HI, followed by stems (2.23 × 10^−5^), and roots (8.51 × 10^−6^). Consumption of leaves and stems could pose a risk approximately ten times higher than consuming roots (Figure 5a). Specifically, the HI value for leaf consumption represented 71.5% of the total HI value of the plant (Figure 5d). The total health risk HI value, a cumulative sum of individual trace element HQ values, was depicted in Figure 5b, totaling 4.76 × 10^−4^ for soil uptake and 1.11 × 10^−4^ for *Scrophularia ningpoensis* Hemsl. plants.

### 3.7. Evaluation of Carcinogenic Risk in Scrophularia ningpoensis Hemsl. Soil and Plant

**Figure 6:**
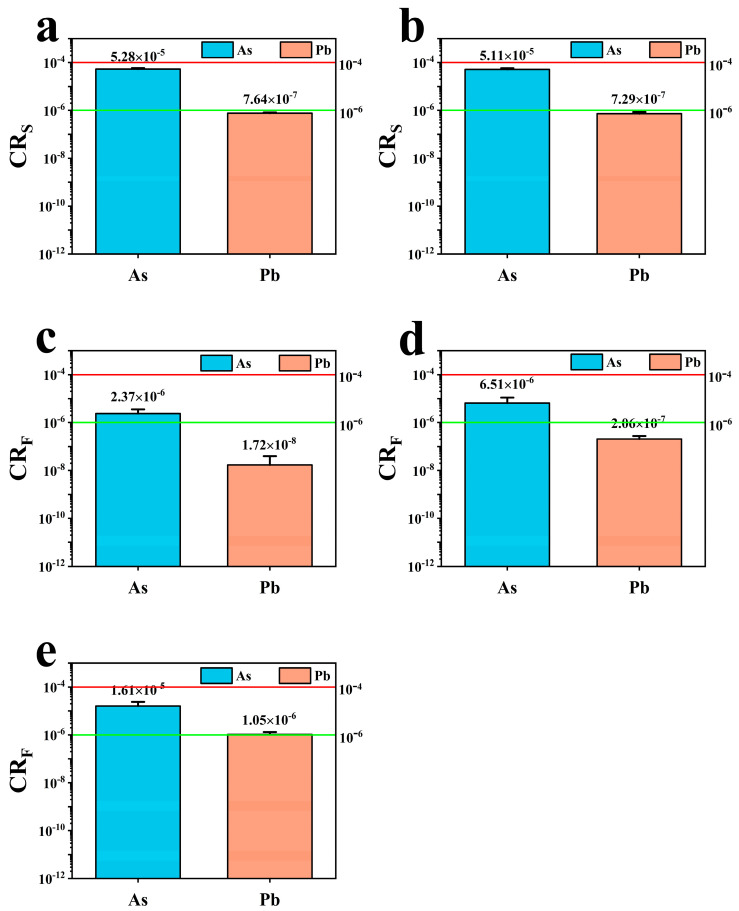
Carcinogenic risk (CR) from direct soil contact and food intake. Note: Figures (**a**–**e**) represent non−root zone soil, root zone soil, *Scrophularia ningpoensis* Hemsl. root, *Scrophularia ningpoensis* Hemsl. stem, and *Scrophularia ningpoensis* Hemsl. leaf, respectively.

Figure 6 presents the carcinogenic risk (CR) values in the soil and plants originating from *Scrophularia ningpoensis* Hemsl. The mean CR_S_ values for element As in the soil of both non-root and root zones were 5.28 × 10^−5^ and 5.11 × 10^−5^, respectively. Although these values were higher than 10^−6^, they remained below the significant threshold of 10^−4^, indicating no substantial carcinogenic risk. However, their values being higher than 10^−6^ warrant attention. For element Pb in the soil of both zones, the mean CR_S_ values were below 10^−6^ across all samples, indicating no carcinogenic risk. Regarding CR_F_ values, for element As in roots, stems, and leaves of *Scrophularia ningpoensis* Hemsl. plants, the mean values were 2.37 × 10^−6^, 6.54 × 10^−6^, and 1.61 × 10^−5^, respectively. These values remained below 10^−4^, signifying no significant carcinogenic risk. Similarly, the mean CR_F_ values of elemental Pb in roots, stems, and leaves were 1.72 × 10^−8^, 2.06 × 10^−7^, and 1.05 × 10^−6^, respectively, all below 10^−4^, indicating no substantial carcinogenic risk existed.

### 3.8. Correlation Analysis of Micronutrient Contents in Non-Root Zone, Root Zone Soils, and Scrophularia ningpoensis Hemsl. Plants (Root, Stem, and Leaf)

**Figure 7:**
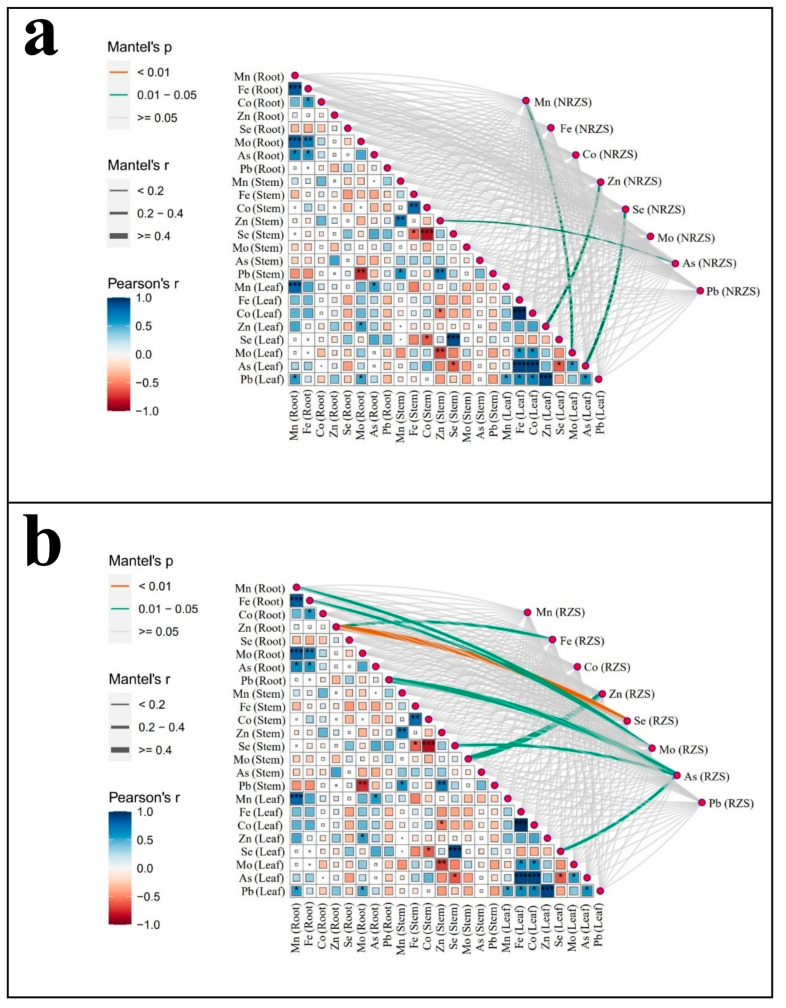
Correlation analysis of trace element contents in non-root zone, root zone soil and *Scrophularia ningpoensis* Hemsl. plants (roots, stems and leaves) of *Scrophularia ningpoensis* Hemsl. Note: Figure (**a**) represents the correlation analysis of trace element contents in soil of non-root zone and *Scrophularia ningpoensis* Hemsl. plants (roots, stems and leaves); Figure (**b**) represents the correlation analysis of trace element contents in soil of root zone and *Scrophularia ningpoensis* Hemsl. plants (roots, stems and leaves). Note: * indicates significant level *p* < 0.05; ** indicates significant level *p* < 0.01; *** indicates significant level *p* < 0.001.

In order to explore the correlation of trace element contents in non-root zone, root zone soils and *Scrophularia ningpoensis* Hemsl. plants (roots, stems and leaves), correlation analyses were carried out, and the results are shown in Figure 7. In non-root zone soils, significant correlations (*p* < 0.05) were found between Mn (NRZS) and Mo (Leaf), Zn (NRZS) and Zn (Leaf), Se (NRZS) and As (Leaf), and As (NRZS) and Zn (Stem) were significantly correlated (*p* < 0.05). In root zone soil, significant correlations (*p* < 0.05) were found between Fe (RZS) and Zn (Root), Zn (RZS) and Mo (Stem), Mo (NRZS) and Mn (Root), As (RZS) and Pb (Root), As (RZS) and Se (Stem), and As (RZS) and Se (Leaf), and Se (RZS) was highly significantly correlated (*p* < 0.01) with Mn (Root). Overall, the correlation between root zone and plant roots, stems and leaves was greater than that between non-root zone and plant roots, stems and leaves.

In this study, eight trace elements were involved, but the importance of these eight trace elements varied greatly in the soil and in different parts of the *Scrophularia ningpoensis* Hemsl. plant. Therefore, we used principal component analysis to screen out some elements with stronger importance. According to the cumulative contribution greater than 70% as the screening index, the screening results were shown in Table 3. Structural equation modeling was then utilized to explore the interrelationships among these elements overall, and the outcomes are presented in Figure 7.

**Figure 8:**
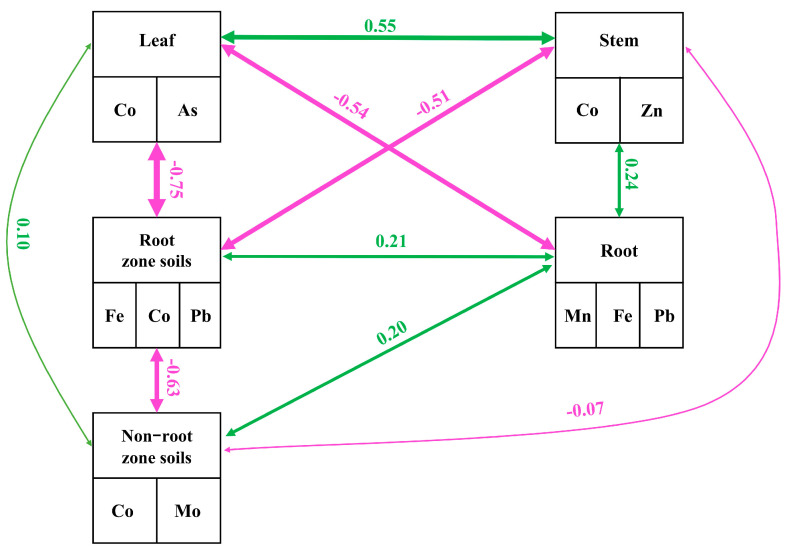
Structural equation modeling of trace element contents in non−root zone, root zone soil, and *Scrophularia ningpoensis* Hemsl. plants (root, stems and leaves). **Note:** Pink represents a negative correlation and green represents a positive correlation.

Significant relationships were observed between non-root zone soils, root zone soils, and plant components (roots, stems, and leaves) in both directions (Figure 8). A notable negative correlation of −0.63 existed between non-root zone soil and root zone soil, signifying a strong antagonistic relationship between these soil elements. The path coefficients between the non-root zone soil and the plant (roots, stems and leaves) were all small although the effects were significant, the largest being the path coefficient between the non-root zone soil and the plant roots (0.20). The path coefficients between root zone soil and plant roots, stems, and leaves were 0.21, −0.51, and −0.75, respectively, and these path coefficients were much higher than the path coefficients between non-root zone soil and plants (roots, stems, and leaves), and this result indicates that the effect of root zone soil on plants is much greater than the effect of non-root zone soil on plants. An interaction was noted among plant roots, stems, and leaves, with a path coefficient of −0.54 between roots and leaves, indicating a strong negative effect. The path coefficient between roots and stems was 0.24, suggesting a moderate positive effect. The path coefficient between stems and leaves was 0.55, signifying a strong positive relationship between these plant elements.

## 4. Discussion

Trace elements found in plants primarily originate from the root zone soil [27]. Variations in trace element contents within plants may stem from the diverse enrichment capacities seen across different trace elements within the same plant and among its various parts. This study revealed that the levels of Mn, Fe, Co, Zn, Mo, As, and Pb in the leaves of *Scrophularia ningpoensis* Hemsl. were notably higher than those in its stems and roots (*p* < 0.05). Furthermore, the enrichment coefficients for Mn, Fe, Co, Zn, Mo, As, and Pb in the leaves were significantly greater compared to the roots and stems of *Scrophularia ningpoensis* Hemsl., signifying a robust enrichment potential in the leaves for these trace elements. This emphasizes the stronger impact of *Scrophularia ningpoensis* Hemsl. leaves on Mn, Fe, Co, Zn, Mo, As, and Pb enrichment. In addition, correlation analysis and structural equation analysis of micronutrient contents of non-root zone soils, root zone soils and different parts of *Scrophularia ningpoensis* Hemsl. showed that root zone soils had a significantly higher effect on *Scrophularia ningpoensis* Hemsl. plants than non-root zone soils. The path coefficients between roots and leaves (−0.54) and stems and leaves (0.55) revealed antagonistic effects between elements in roots and leaves and synergistic effects between elements in stems and leaves [28]. This variance might contribute significantly to the different trace element contents observed in distinct plant parts. Studies have highlighted that the relationship between elements in soil and plant parts often varies, leading to diverse elemental expressions in both the soil and the plant [29].

Given the potential for certain trace elements to accumulate in the edible parts of crops via the food chain [30,31], increased scrutiny of trace element content and health risks in various agricultural products within the study area is crucial. Findings from this study revealed Pi values exceeding 1.0 in three samples each of non-root and root zone soils, with contaminated samples accounting for 21.4%. Meanwhile, the combined contamination index PN of non-root and root zone soils fell between 0.7 and 1.0, indicating an alerting contamination level in the soil of the *Scrophularia ningpoensis* Hemsl. production area, characterized by a certain degree of micronutrient contamination. Notably, none of the samples, whether soil or plant, produced a health risk index (HI) higher than 1. This outcome markedly contrasts with similar studies; for instance, Tang et al. [32] reported a median HI of 1.40, Li et al. [33] found a HI of 2.45, Jiang et al. [34] recorded an HI of 3.62, and Antoniadis et al. [35] observed an HI of 1.62. These studies shared a common trait: they originated from highly contaminated areas. Conversely, our study was conducted in a Chinese herbal medicine plantation within an agricultural land where the primary sources of pollution were agricultural irrigation and fertilizer use, as opposed to the severe pollution sources found in heavily polluted areas such as industrial zones, coal mining, and smelters. These sources of pollution typically generate less contamination compared to industrial pollution sources.

The carcinogenic risk (CR) values within the soil-*Scrophularia ningpoensis* Hemsl. system all remained below 10^−4^, indicating no significant carcinogenic risk. However, it is essential to note that the CR_S_ associated with elemental As in the soil-*Scrophularia ningpoensis* Hemsl. system surpassed 10^−6^, signaling a certain level of carcinogenic risk. Notably, our study identified that the CR_S_ of the root zone soils in the *Scrophularia ningpoensis* Hemsl. origin were consistently lower than those of the non-root zone soils. This trend might be attributed to the substantial accumulation of soil trace elements within most plants, possibly contributing to the lower CR_S_ observed in root zone soils compared to non-root zone soils. Additionally, our investigation revealed variations in CR across different parts of the *Scrophularia ningpoensis* Hemsl. plant. The highest carcinogenic risk was observed in the edible leaves, followed by the stems, with the lowest risk found in the roots.

## 5. Conclusions

Variations were evident in the trace element content characteristics within the soil of *Scrophularia ningpoensis* Hemsl. origin, consistently showing higher levels in non-root zone soil compared to the root zone. Among the different segments of the *Scrophularia ningpoensis* Hemsl. plant, except for Se, leaves exhibited the highest trace element content, succeeded by stems, while roots displayed the lowest content. Correlation analysis and structural equation analysis highlighted the notable influence of root zone soil on the plant, outweighing the impact of non-root zone soil. This analysis also unveiled synergistic or antagonistic interactions among trace elements in the plant’s roots, stems, and leaves. Moreover, the evaluation based on the single-factor pollution index (Pi) indicated a certain level of soil pollution in the *Scrophularia ningpoensis* Hemsl. origin. Particularly, the highest average Pi value was recorded for Pb, reaching 0.94 and 0.89 in the non-root and root zones, respectively. The combined Nemerow pollution index (PN) for both zones fell within the warning range. Evaluation of non-carcinogenic risks highlighted that the consumption of *Scrophularia ningpoensis* Hemsl. leaves and stems may potentially pose a non-carcinogenic risk (HI) and carcinogenic risk (CR) more than tenfold higher than that from roots.

## Figures and Tables

**Table 1:** Bioconcentration factor of roots, stems, and leaves of *Scrophularia ningpoensis* Hemsl.

Place	Statistics	Mn	Fe	Co	Zn	Se	Mo	As	Pb
Root	Maximum	0.04	0.02	0.05	0.05	0.09	0.09	0.02	0.02
	Minimum	0.01	0.00	0.01	0.01	0.00	0.01	0.00	0.00
	Mean	0.03 ^b^	0.01 ^b^	0.02 ^b^	0.03 ^b^	0.02 ^a^	0.03 ^b^	0.01 ^c^	0.00 ^c^
	Standard Deviation	0.01	0.00	0.01	0.01	0.03	0.02	0.00	0.01
Stem	Maximum	0.09	0.03	0.03	0.07	0.01	0.03	0.04	0.09
	Minimum	0.02	0.01	0.01	0.02	0.00	0.00	0.00	0.00
	Mean	0.04 ^b^	0.02 ^b^	0.02 ^b^	0.04 ^b^	0.00 ^b^	0.00 ^b^	0.02 ^b^	0.05 ^b^
	Standard Deviation	0.02	0.01	0.01	0.02	0.00	0.01	0.01	0.02
Leaf	Maximum	0.29	0.10	0.11	0.10	0.01	0.22	0.10	0.44
	Minimum	0.03	0.02	0.02	0.05	0.00	0.01	0.02	0.16
	Mean	0.17 ^a^	0.05 ^a^	0.05 ^a^	0.07 ^a^	0.00 ^b^	0.07 ^a^	0.05 ^a^	0.25 ^a^
	Standard Deviation	0.08	0.03	0.03	0.02	0.00	0.07	0.02	0.09

Note: Different lowercase letters indicate significantly different enrichment coefficients of the same element in roots, stems, and leaves (*p* < 0.05).

**Table 2:** Associated HQ Values in Soil–*Scrophularia ningpoensis* Hemsl. System.

HQ	Place	Statistics	Mn	Fe	Co	Zn	Se	Mo	As	Pb
HQ_S_	Non-root zone	Maximum	1.61 × 10^−5^	8.90 × 10^−5^	2.35 × 10^−5^	1.86 × 10^−8^	1.16 × 10^−6^	6.14 × 10^−7^	1.51 × 10^−4^	2.82 × 10^−5^
		Minimum	5.49 × 10^−6^	5.55 × 10^−5^	1.06 × 10^−5^	6.72 × 10^−9^	5.88 × 10^−7^	1.98 × 10^−7^	1.04 × 10^−4^	2.13 × 10^−5^
		Mean	1.06 × 10^−5^	6.93 × 10^−5^	1.63 × 10^−5^	1.35 × 10^−8^	9.10 × 10^−7^	4.20 × 10^−7^	1.17 × 10^−4^	2.57 × 10^−5^
		Standard Deviation	3.22 × 10^−6^	1.08 × 10^−5^	3.35 × 10^−6^	4.40 × 10^−9^	1.72 × 10^−7^	1.41 × 10^−7^	1.41 × 10^−5^	1.88 × 10^−6^
	Root zone	Maximum	1.67 × 10^−5^	8.77 × 10^−5^	2.28 × 10^−5^	2.25 × 10^−8^	1.32 × 10^−6^	5.88 × 10^−7^	1.54 × 10^−4^	2.93 × 10^−5^
		Minimum	5.90 × 10^−6^	5.67 × 10^−5^	1.20 × 10^−5^	9.34 × 10^−9^	4.05 × 10^−7^	1.93 × 10^−7^	9.28 × 10^−5^	1.28 × 10^−5^
		Mean	1.07 × 10^−5^	6.84 × 10^−5^	1.69 × 10^−5^	1.56 × 10^−8^	9.28 × 10^−7^	3.66 × 10^−7^	1.14 × 10^−4^	2.45 × 10^−5^
		Standard Deviation	3.61 × 10^−6^	1.10 × 10^−5^	3.69 × 10^−6^	3.77 × 10^−9^	2.36 × 10^−7^	1.31 × 10^−7^	1.56 × 10^−5^	4.01 × 10^−6^
HQ_F_	Root	Maximum	2.81 × 10^−6^	6.36 × 10^−6^	3.80 × 10^−6^	4.33 × 10^−9^	4.24 × 10^−7^	3.11 × 10^−7^	3.47 × 10^−6^	2.49 × 10^−6^
		Minimum	8.50 × 10^−7^	2.29 × 10^−6^	7.88 × 10^−7^	1.15 × 10^−9^	0.00	1.85 × 10^−8^	7.79 × 10^−7^	0.00
		Mean	1.46 × 10^−6^	3.63 × 10^−6^	1.64 × 10^−6^	2.81 × 10^−9^	1.24 × 10^−7^	7.35 × 10^−8^	1.58 × 10^−6^	5.77 × 10^−7^
		Standard Deviation	4.92 × 10^−7^	1.15 × 10^−6^	7.22 × 10^−7^	9.48 × 10^−10^	1.62 × 10^−7^	7.23 × 10^−8^	7.79 × 10^−7^	7.62 × 10^−7^
	Stem	Maximum	3.38 × 10^−6^	1.22 × 10^−5^	3.03 × 10^−6^	6.27 × 10^−9^	7.60 × 10^−8^	7.31 × 10^−8^	9.76 × 10^−6^	9.49 × 10^−6^
		Minimum	1.51 × 10^−6^	3.81 × 10^−6^	1.03 × 10^−6^	1.40 × 10^−9^	0.00	0.00	2.17 × 10^−7^	3.75 × 10^−7^
		Mean	2.45 × 10^−6^	7.33 × 10^−6^	2.08 × 10^−6^	3.41 × 10^−9^	1.86 × 10^−8^	1.11 × 10^−8^	4.34 × 10^−6^	6.93 × 10^−6^
		Standard Deviation	6.59 × 10^−7^	2.67 × 10^−6^	5.98 × 10^−7^	1.35 × 10^−9^	2.76 × 10^−8^	2.20 × 10^−8^	3.00 × 10^−6^	2.36 × 10^−6^
	Leaf	Maximum	1.68 × 10^−5^	5.00 × 10^−5^	1.10 × 10^−5^	8.95 × 10^−9^	6.37 × 10^−8^	4.00 × 10^−7^	2.29 × 10^−5^	5.28 × 10^−5^
		Minimum	3.26 × 10^−6^	6.80 × 10^−6^	2.35 × 10^−6^	4.58 × 10^−9^	0.00	1.15 × 10^−8^	4.79 × 10^−6^	2.23 × 10^−5^
		Mean	9.57 × 10^−6^	1.90 × 10^−5^	4.87 × 10^−6^	6.36 × 10^−9^	1.35 × 10^−8^	1.41 × 10^−7^	1.08 × 10^−5^	3.52 × 10^−5^
		Standard Deviation	3.82 × 10^−6^	1.31 × 10^−5^	2.79 × 10^−6^	1.39 × 10^−9^	2.37 × 10^−8^	1.31 × 10^−7^	5.09 × 10^−6^	8.55 × 10^−6^

**Table 3:** Principal component analysis screening results.

Place	Trace Element
Non-root zone soils	Co, Mo
Root zone soils	Fe, Co, Pb
Root	Mn, Fe, Pb
Stem	Co, Zn
Leaf	Co, As

## Data Availability

The data used in this study are all from experiments. The corresponding author can be contacted if necessary.

## Appendix A

Appendix A values for health risk assessment used in this work concerning oral reference doses (RfD_0_), tolerable daily intake (TDI) in μg of potentially toxic elements per kg of bodyweight per day related to non-carcinogenic health risk, and oral slope factor (OSF; unitless) related to carcinogenic health risk [26].

**Table A1:** Parameter values related to health risks.

	RfD_0_	TDI	OSF
As	0.3	1	1.5
Co	0.3	1.4	NA
Fe	700	NR	NA
Mn	140	NR	NA
Mo	5	10	NA
Pb	3.5	NR	0.0085
Se	5	4	NA
Zn	300	500	NA

NR = Non-reported. When a value for a given element is non-reported, we utilized the RfD_0_ of that same element instead. NA = Non-applicable.
